# Mechanisms Underlying Aquaporin-4 Subcellular Mislocalization in Epilepsy

**DOI:** 10.3389/fncel.2022.900588

**Published:** 2022-06-06

**Authors:** Jenny I. Szu, Devin K. Binder

**Affiliations:** Division of Biomedical Sciences, School of Medicine, University of California, Riverside, Riverside, CA, United States

**Keywords:** aquaporin-4, epilepsy, astrocytes, subcellular localization, seizures

## Abstract

Epilepsy is a chronic brain disorder characterized by unprovoked seizures. Mechanisms underlying seizure activity have been intensely investigated. Alterations in astrocytic channels and transporters have shown to be a critical player in seizure generation and epileptogenesis. One key protein involved in such processes is the astrocyte water channel aquaporin-4 (AQP4). Studies have revealed that perivascular AQP4 redistributes away from astrocyte endfeet and toward the neuropil in both clinical and preclinical studies. This subcellular mislocalization significantly impacts neuronal hyperexcitability and understanding how AQP4 becomes dysregulated in epilepsy is beginning to emerge. In this review, we evaluate the role of AQP4 dysregulation and mislocalization in epilepsy.

## Introduction

Epilepsy is a chronic brain disorder characterized by spontaneous recurrent (two or more) seizures (Fisher et al., 2014). It is a global health problem affecting more than 65 million people worldwide (Clossen and Reddy, 2017). The incidence of epilepsy is 61.44 per 100,000 persons-year (Beghi and Hesdorffer, 2014; Fiest et al., 2017). The economic burden associated with this disease is staggering with total direct healthcare costs ranging between $9.6 – 12.5 billion per year in the United States (Lekoubou et al., 2018). Furthermore, quality of life is profoundly impacted in patients living with epilepsy. Some individuals are unable to function independently, have lower rates of employment, and experience difficulties with social engagement (Yogarajah and Mula, 2019). Epilepsy is also comorbid with several conditions including depression, diabetes, asthma, and stroke (Ottman et al., 2011).

Astrocytes are the most abundant cell type in the central nervous system (CNS) and are involved in key functions such as maintaining ionic homeostasis (Simard and Nedergaard, 2004), regulating the blood-brain barrier (BBB) (Abbott, 2002), and providing metabolic support (Brown and Ransom, 2007). Because astrocytes display such diverse functions, any changes in astrocyte molecules significantly impact epileptogenesis (Binder and Steinhäuser, 2021). Astrocytes express a wide array of ion channels, neurotransmitter receptors, and transporters all which detect and respond to neuronal activity (Coulter and Steinhäuser, 2015; Steinhäuser et al., 2016). For instance, glutamate transporter-1 (GLT-1) aids in clearance of glutamate, the most abundant excitatory neurotransmitter from the synaptic cleft (Danbolt, 2001). Kir4.1, an inwardly-rectifying potassium channel, maintains K^+^ homeostasis by either net uptake or spatial buffering (Orkand et al., 1966; Olsen and Sontheimer, 2008; Bay and Butt, 2012; Macaulay and Zeuthen, 2012). Na^+^-K^+^-2Cl^−^ cotransporter (NKCC1) regulates GABA receptors by maintaining intracellular Cl^−^ levels (Dzhala et al., 2005; Liu et al., 2020). These astrocytic molecules, in addition to others, work in concert to prevent excessive neuronal firing.

Aquaporin-4 (AQP4) is a member of the aquaporin (AQP) family of small, hydrophobic, integral membrane proteins (Verkman, 2005). This water-selective transporter is heterogeneously expressed throughout the CNS (Hubbard et al., 2015) and is mainly found in regions of fluid transport such as the BBB and the brain-cerebrospinal fluid (CSF) barrier (Verkman et al., 2006). AQP4 is primarily expressed by astrocytes and is heavily enriched at their endfeet where it aids in bidirectional transport of water in response to osmotic gradients (Verkman, 2005; Tait et al., 2008; Papadopoulos and Verkman, 2013). The simple function of moving water has created diverse roles of AQP4. For instance, AQP4 facilitates astrocyte migration by regulating osmotic water flux across the cell membrane by increasing water permeability in the front end of the cell (Papadopoulos et al., 2008). It is not surprising that another critical function of AQP4 is to maintain water homeostasis. Tissue swelling due to accumulation of excess water commonly observed in brain edema and stroke (Papadopoulos and Verkman, 2007; Zador et al., 2009) can have detrimental outcomes. AQP4 has been proposed to play a role in the glymphatic system where it facilitates the clearance of extracellular solutes (Iliff et al., 2012, 2013; Plog et al., 2015; Xu et al., 2015; Lundgaard et al., 2017; Mestre et al., 2018; Iliff and Simon, 2019; Salman et al., 2022a). While this concept has generated much enthusiasm, it remains highly controversial (Abbott, 2002; Hladky and Barrand, 2014; Spector et al., 2015; Smith et al., 2017; Abbott et al., 2018; Smith and Verkman, 2018; Hablitz et al., 2020).

## Modulation of Seizure Activity By AQP4

It is remarkable that a simple water channel such as AQP4 plays such crucial roles in seizure activity and epilepsy. Indeed, space within the brain is strictly regulated by maintaining water, ion, and neurotransmitter concentrations. But what are the mechanisms that connect water transport to neuronal hyperactivity? The most fundamental explanation is that increases in cellular volume have a dramatic influence on neuronal activity. In fact, it has long been established that neuronal excitability is incredibly sensitive to osmolarity and changes in the extracellular space (ECS) (Dudek et al., 1990; Andrew, 1991; Schwartzkroin et al., 1998). Reductions in ECS increases concentrations of extracellular ions and neurotransmitters and intensifies ephaptic interactions of closely interacting neurons resulting in more synchronous firing and bursting activity (Andrew et al., 1989; Mccormick and Contreras, 2001). Thus, activity-dependent volume changes of the ECS are tightly regulated by AQP4 and represent a key element of seizure and epileptiform activity generation. Below, we highlight a few molecules that are thought to interact with AQP4 in modulating neuronal activity.

### AQP4 and Kir4.1

The subcellular distribution of AQP4 mimics that of Kir4.1, suggesting that they may work together as a functional unit (Nagelhus et al., 2004; Smith and Verkman, 2015). After neuronal activation, increases in extracellular K^+^ enhances intracellular osmolarity which drives water into cells via AQP4 resulting in astrocyte swelling (Andrew and Macvicar, 1994; Risher et al., 2009; Murphy et al., 2017a). Therefore, altered AQP4 expression could potentially impact potassium reuptake by Kir4.1. Studies have demonstrated that mice lacking AQP4 exhibited an increase in electrographic seizure threshold and a significantly longer electrical stimulation-induced seizure duration (Binder et al., 2006). The increased seizure threshold may be attributed to an expanded ECS volume that was previously observed in AQP4 knockout (AQP4 KO) mice (Binder et al., 2004) in which stronger stimuli may be required to overcome the enhanced ECS to initiate a seizure. On the other hand, the prolonged seizure duration may be correlated to deficits in K^+^ reuptake. Indeed, impaired K^+^ kinetics was observed in AQP4 KO mice in which a delay in the rise to peak K^+^ and decay to baseline K^+^ was detected following *in vivo* cortical stimulation (Binder et al., 2006). Dysfunctional K^+^ reuptake were also observed in AQP4-deficient mice where antidromic stimulation of CA1 neurons led to small increases and slower recovery of [K^+^]_o_. Interestingly, however, these mice displayed enhanced spatial K^+^ buffering which may be due to the increased gap junction coupling of astrocytes (Strohschein et al., 2011). Computational modeling further supported these findings where it showed that AQP4 deficiency led to reduced water permeability in astrocytes as well as impaired K^+^ accumulation and reuptake (Jin et al., 2013).

### AQP4 and GLT-1

Astrocytic GLT-1 is the primary transporter responsible for glutamate clearance (Danbolt, 2001) and excess levels of extracellular glutamate not only contribute to recurrent seizures but can also affect neuronal signaling and network connectivity long-term (Eid et al., 2008; Barker-Haliski and White, 2015). Therefore, downregulation or lack of GLT-1 can severely impact glutamate homeostasis exacerbating neuronal hyperactivity. Decreased levels of GLT-1 have been reported in both clinical (Mathern et al., 1999; Proper et al., 2002) and preclinical (Tanaka et al., 1997; Hubbard et al., 2016; Sugimoto et al., 2018; Peterson and Binder, 2019) studies of epilepsy. Furthermore, colocalization of AQP4 with GLT-1 have also been observed (Hinson et al., 2008; Lee et al., 2013; Yang et al., 2013; Mogoanta et al., 2014). Previous findings have reported reductions in GLT-1 and subsequent glutamate clearance in mice lacking AQP4 (Zeng et al., 2007; Wu et al., 2008; Li et al., 2012; Yang et al., 2013). The coexpression of these two proteins have sparked speculation that they may be part of the same complex and be involved in similar signal transductions. Interestingly however, a physical interaction between AQP4 and GLT-1 was not detected in healthy wild-type (WT) mice and impairments in GLT-1 was not observed in AQP4-null mice (Hubbard and Binder, 2017). Moreover, AQP4 and GLT-1 were found to be differentially regulated in a model of TLE (Hubbard et al., 2016).

### AQP4 and mGluR5

Metabotropic glutamate receptors (mGluRs) are G-protein-coupled receptors and are key modulators of cell excitability and synaptic transmission (Peterson and Binder, 2020). mGluR5 is predominantly expressed by developing astrocytes (Sun et al., 2013) and has been shown to interact with AQP4 (Illarionova et al., 2010). Glutamate increases astrocyte water permeability and astrocyte swelling is mediated in part by mGluR5 activation (Gunnarson et al., 2008; Illarionova et al., 2010; Shi et al., 2017). mGluR5 dysregulation has been reported in epilepsy (Aronica et al., 2000, 2003; Kandratavicius et al., 2013) and its expression levels may serve as a biomarker of active epilepsy. Mice that displayed persistent mGluR5 expression during the latent period eventually developed epilepsy compared those that only exhibited transiently elevated mGluR5 expression levels. Additionally, mice lacking mGluR5 displayed slowed glutamate clearance during high-frequency stimulation (Umpierre et al., 2019) which is consistent with human findings in which lower mGluR5 expression correlates with increased seizure frequency (Kandratavicius et al., 2013). It appears that mGluR5 upregulation may serve as a compensatory mechanism during epileptogenesis which aligns with the upregulation of AQP4 observed in chronic epilepsy (Das et al., 2012; Hubbard et al., 2016). Therefore, it is likely that mGluR5 KO mice may exhibit decreased levels of AQP4 exerting pro-epileptogenic effects.

### AQP4 and gap Junctions

A unique feature of astrocytes is their ability to organize into large networks that are enabled by extensive gap junction coupling composed of connexin 30 (Cx30) and 43 (Cx43) (Anders et al., 2014). The coupling of astrocytes not only allows for intercellular communication but also functions to clear extracellular K^+^ (spatial K^+^ buffering) and glutamate and deliver neurometabolites (Giaume et al., 2010). Loss of astrocyte coupling can further impact astrocyte functions that is essential for regulating neuronal hyperexcitability (Steinhäuser et al., 2012; Boison and Steinhäuser, 2018). In the sclerotic hippocampus of patients with MTLE, almost complete loss of astrocytes and gap function coupling were found. In a mouse model of MTLE, deficits in astrocyte coupling and K^+^ clearance were detected within 4 hours of kainic acid (KA) induced status epilepticus (SE) (Bedner et al., 2015). Although there is no physical interaction between the two, studies have shown that AQP4 and connexins may be interdependent. For instance, AQP4 KO mice exhibited enhanced gap junction coupling (Strohschein et al., 2011) and an upregulation of Cx43 (Katoozi et al., 2017). Additionally, deletion of Cx43 and Cx30 resulted in significant reductions of perivascular AQP4 expression as well as decreased AQP4 mRNA and protein levels (Katoozi et al., 2020). Therefore, impairments in astrocyte coupling leading to AQP4 dysregulation can have profound effects on other molecules (such as those mentioned above) which ultimately bolsters seizure activity.

## AQP4 Dysregulation In Epilepsy

Epilepsy is a highly complex and dynamic neurological disorder that involves extensive interplay between AQP4 and other proteins. It is well established that deficits in water homeostasis, as regulated by AQP4, are associated with neuronal hyperexcitability and that cell volume and ion regulation are critical in both seizure activity and epilepsy. We have briefly discussed at how AQP4 deficiencies influences neuronal activity. Below, we examine further how AQP4 dysregulation, particularly its subcellular mislocalization, may contribute to epileptogenesis.

### AQP4 Expression

To begin to understand how AQP4 mislocalization affects epilepsy, it is necessary to recognize its baseline expression pattern. While it is clear that AQP4 is abundantly expressed at astrocyte endfeet (Nielsen et al., 1997; Badaut et al., 2000, 2002; Nagelhus et al., 2004) studies have also developmentally-regulated and region-specific expression of AQP4 (Hsu et al., 2011; Hubbard et al., 2015). In the mouse brain, AQP4 was found throughout the forebrain, subcortical areas, and brainstem with highest and lowest AQP4 protein levels in the cerebellum and hippocampus, respectively (Hubbard et al., 2015). Furthermore, AQP4 displayed laminar specificity in the hippocampus with highest expression in the CA1 stratum lacunosum-moleculare and molecular layer of the dentate gyrus (Hsu et al., 2011). Involvement of the hippocampus in seizure generation and epilepsy are well documented and hippocampal sclerosis is a hallmark of temporal lobe epilepsy (TLE) as well as other epilepsy syndromes (Thom, 2014). Thus, changes in hippocampal AQP4 in epilepsy may have a powerful impact on seizure outcome. Nevertheless, it is crucial to note that changes in expression patterns (up- or downregulation) of AQP4 as well as its surface localization are two separate concepts and that each outcome does not necessarily accompany the other. Indeed, studies have reported increased surface localization of AQP4 despite no changes in total protein levels (Ren et al., 2013; Salman et al., 2017a). Below, we describe reports on AQP4 expression levels and its redistribution in epilepsy.

### AQP4 Mislocalization in Epilepsy

AQP4 dysregulation has been observed in rodent models of TLE. Hippocampal AQP4 was significantly downregulated 1 day after induction of SE with KA with partial recovery at chronic time points (Lee et al., 2012; Hubbard et al., 2016). Surprisingly, no overall significant changes in Kir4.1, the main Kir subtype in the hippocampus (Seifert et al., 2009), were observed (Lee et al., 2012). Rather, a trending upregulation of Kir4.1 was detected, specifically in reactive astrocytes in the CA1 region indicating that APQ4 and Kir4.1 are uniquely regulated during epileptogenesis (Lee et al., 2012). Similar findings were also observed in patients with TLE where AQP4 mRNA and protein levels were significantly increased in the sclerotic hippocampi compared to the neocortex. Similarly, no differences in Kir4.1 mRNA was detected (Salman et al., 2017b). On the other hand, a slight increase in GLT-1 was observed 1 day after SE followed by significant reductions in expression levels at 4 and 7 days post SE followed by partial recovery by day 30 (Hubbard et al., 2016). Like Kir4.1, it appears that GLT-1 also display distinct patterns or regulation during the development of epilepsy. Similar reductions in AQP4 levels were also observed in the piriform cortex and hippocampus of rats after induction of SE using pilocarpine as early as 12 hours after SE. Most notably, an “AQP4-deleted” area was detected in the piriform cortex but not hippocampus of animals that experienced SE (Kim et al., 2010). The early decrease in AQP4 points to the fact that AQP4 dysregulation begins early in the process of epileptogenesis.

The findings reported above represents an overall reduction in AQP4 in select regions of the brain. Indications of subcellular mislocalization of AQP4 may provide a better mechanistic view of how AQP4 dysregulation contributes to a pro-epileptogenic effect. Interestingly, hippocampal AQP4 expression was found to be mislocalized in rats following SE induced by systemic KA. More specifically, immunogold labeling revealed reductions in AQP4 density in the adluminal endfeet of astrocytes during the latent phase (before the onset of chronic epileptic seizures) (Alvestad et al., 2013). Subcellular redistribution of AQP4 has also been observed in a mouse model of posttraumatic epilepsy (PTE). Unlike human and rodent models of TLE, overall AQP4 levels in the hippocampus were unchanged over the course of epileptogenesis after induction of a moderate – severe traumatic brain injury (Szu et al., 2020). Surprisingly, a significant increase in cortical and hippocampal AQP4 was detected in the subset of mice that developed PTE compared to those that did not develop PTE. Moreover, confocal microscopy revealed that AQP4 was now largely expressed in the soma and major processes of astrocytes in mice with PTE while perivascular AQP4 was maintained in mice that did not develop PTE (Szu et al., 2020).

Several human studies of epilepsy have reported altered expression of AQP4 as well. In postmortem human samples, significant upregulation of AQP4 immunoreactivity was observed in the sclerotic hippocampi of patients with TLE compared to non-scleortic hippocampi or controls (Lee et al., 2004; Das et al., 2012). AQP4 transcript levels were also markedly upregulated in the sclerotic hippocampi. A positive correlation between AQP4 and GFAP mRNA was detected indicating that enhanced levels of AQP4 is presumably attributed to an increase in reactive astrocytes (Lee et al., 2004). Similar findings were also observed in patients with mesial TLE (MTLE). Compared with non-MTLE patients, AQP4 levels were significantly increased by an astounding 360%. Similar to the animal studies, the profound increase in AQP4 associated with AQP4 mislocalization. In fact, immunogold labeling of AQP4 found a 44% reduction of perivascular AQP4 in the MTLE hippocampi compared the non-MTLE hippocampi. However, a labeling of AQP4 in the neuropil was 173% greater in the MTLE hippocampi (Eid et al., 2005). These findings suggest that AQP4 is redistributed in TLE since at baseline AQP4 is more enriched at astrocytic endfeet surrounding blood vessels (Verkman, 2005).

## Mechanisms Underlying AQP4 Subcellular Redistribution

While it has been demonstrated that AQP4 is mislocalized in epilepsy, the mechanisms underlying this phenomenon remain to be determined. However, understanding how AQP4 is regulated may provide clues on how it becomes perturbed during disease progression.

### AQP4 Isoforms Regulate Astrocyte Processes

There are two major isoforms of AQP4 produced by alternative splicing: the long M1 isoform with translation initiation at Met-1 and the short M23 isoform with translation initiation at Met-23 (Furman et al., 2003; Verkman et al., 2011). Together, they form the supramolecular structures called orthogonal arrays of particles (OAPs) (Verkman et al., 2011). No OAPs were observed in mice lacking AQP4, confirming that AQP4 is required for formation of OAPs (Verbavatz et al., 1997). Freeze-fracture electron microscopy further revealed that Chinese hamster ovary (CHO) cells transfected with either M1, M23, or M1 + M23 isoforms have opposing effects on AQP4 intramembrane organization. Specifically, M23 isoform assemble into sizeable OAPs, indicative of stable configuration. M1 isoforms, on the other hand, are mainly organized as individual AQP4 tetramers. When cells were transfected with both isoforms, M1 and M23 coexpressed as both hetero- and homotetramers, suggesting that these two isoforms interfere with one another (Furman et al., 2003). In fact, early studies have demonstrated that AQP4 heterotetramers are formed by overlapping M1 and M23 isoforms. Furthermore, the M23 isoform is abundantly expressed at the perivascular astrocyte endfeet where M1 isoform also colocalizes (Neely et al., 1999). These findings indicates that AQP4 OAPs are formed by the co-assembly of M1 and M23 isoforms (Crane et al., 2009).

Each AQP4 isoform exhibits distinct membrane dynamics that may explain how AQP4 becomes dysregulated in epilepsy. Tracking of single AQP4 labeled with quantum dots revealed that the M1 isoform diffused freely while the M23 isoform was relatively immobile (Crane et al., 2008; Smith et al., 2014). Recently, studies using superresolution, single-molecule, and calcium imaging of hippocampal astrocytes revealed that AQP4-M23 regulate astrocyte motility and favors glutamate synapse activity. Indeed, AQP4-M23 clusters were strongly expressed at astrocytic processes near glutamatergic synapses (Crane et al., 2008). Additionally, localization of AQP4 is dependent on aggregation properties of M1-AQP4 and M23-AQP4 and likely exhibit different functional roles. For instance, because M1-AQP4 single tetramers are able to diffuse throughout the plasma membrane they may be required for lamellipodial extension (Smith et al., 2014) during astrocyte migration supported by ion transport and AQP4-mediated water influx (Saadoun et al., 2005). Because of their ability to form stable OAPs, M23-AQP4 mainly function in polarization of AQP4 at astrocyte endfeet (Smith et al., 2014).

Because of their distinct localization, surface dynamics, and organization of AQP4-M23, it is plausible that AQP4-M23 may be altered under certain pathological conditions. In fact, binding of neuromyelitis optica IgG to AQP4 directly altered surface diffusion of AQP4-M23 ultimately decreasing astrocyte process motility and glutamate synapse transmission (Ciappelloni et al., 2019). This framework falls in line with the displacement of AQP4 expression in the TLE and PTE studies mentioned above. It is likely that during epileptogenesis that the immobilization of AQP4-M23 to the perivascular endfeet is lost and that regulation of astrocytic process motility to tune basal glutamatergic transmission is severely impaired. This would ultimately result in increased extracellular glutamate concentration and astrocyte swelling allowing for epileptic seizures to occur. In fact, rats treated with KA exhibited a significant increase in M1-AQP4 during the latent phase, however both M23-AQP4 and M1-AQP4 were significantly upregulated in the chronic phase of TLE compared to controls (Alvestad et al., 2013). It is plausible that enhanced M1-AQP4 levels observed during the latent phase reflects redistribution of AQP4 from the perivascular membrane toward the neuropil. However, during the chronic phase, increased M23-AQP4 may suggest a possible compensatory mechanism. While KA-injected mice exhibited a decrease in AQP4 at the later time points, it remains to be determined which AQP4 isoform is involved (Lee et al., 2012; Hubbard et al., 2016). However, in the human studies (Eid et al., 2005) as well as the mouse model of PTE (Szu et al., 2020) where there is a clear observation of AQP4 subcellular mislocalization, it is likely that the increased AQP4 expression in the neuropil represents the M1-AQP4 isoform.

### α-Syntrophin Anchoring of AQP4

Loss of AQP4 polarity in epilepsy also may be correlated to the loss of its anchoring protein. Studies have found that perivascular AQP4 enrichment is associated with the adaptor protein α-syntrophin (α-syn) (Amiry-Moghaddam et al., 2004a). Moreover, it was postulated that tethering of perivascular AQP4 is due to its unique binding with PSD95-Discs large-ZO1 (PDZ) domain of α-syn (Neely et al., 2001). Indeed, an intermolecular interaction between PDZ binding domain of AQP4 was discovered to secure OAPs to the cytoplasmic side of the membrane, reducing M23-AQP4 diffusion (Crane et al., 2008). Furthermore, studies found that while the total levels of AQP4 appeared normal in α-syn null mice, striking reductions in perivascular AQP4 was detected (Neely et al., 2001; Amiry-Moghaddam et al., 2003, 2004b). More surprising was the fact that AQP4 levels were significantly enhanced in non-endfeet membranes of mice lacking α-syn (Amiry-Moghaddam et al., 2004b). These findings indicate deletion in α-syn results in the mislocalization of AQP4 rather than a net loss of AQP4. After KA-induced SE in rats, a significant reduction in hippocampal α-syn was detected in the latent phase, mirroring the significant loss of AQP4. This was associated with the marked increase in M1-AQP4 detected (Alvestad et al., 2013). Significant reductions in hippocampal α-syn was also detected in human patients with TLE (Das et al., 2012; Heuser et al., 2012) that was concurrent with significant increases in AQP4 (Das et al., 2012). Together, these findings indicate that loss of the anchoring protein α-syn results in loss of perivascular AQP4 polarization in epilepsy.

### Posttranslational Modification of AQP4

**Figure 1:**
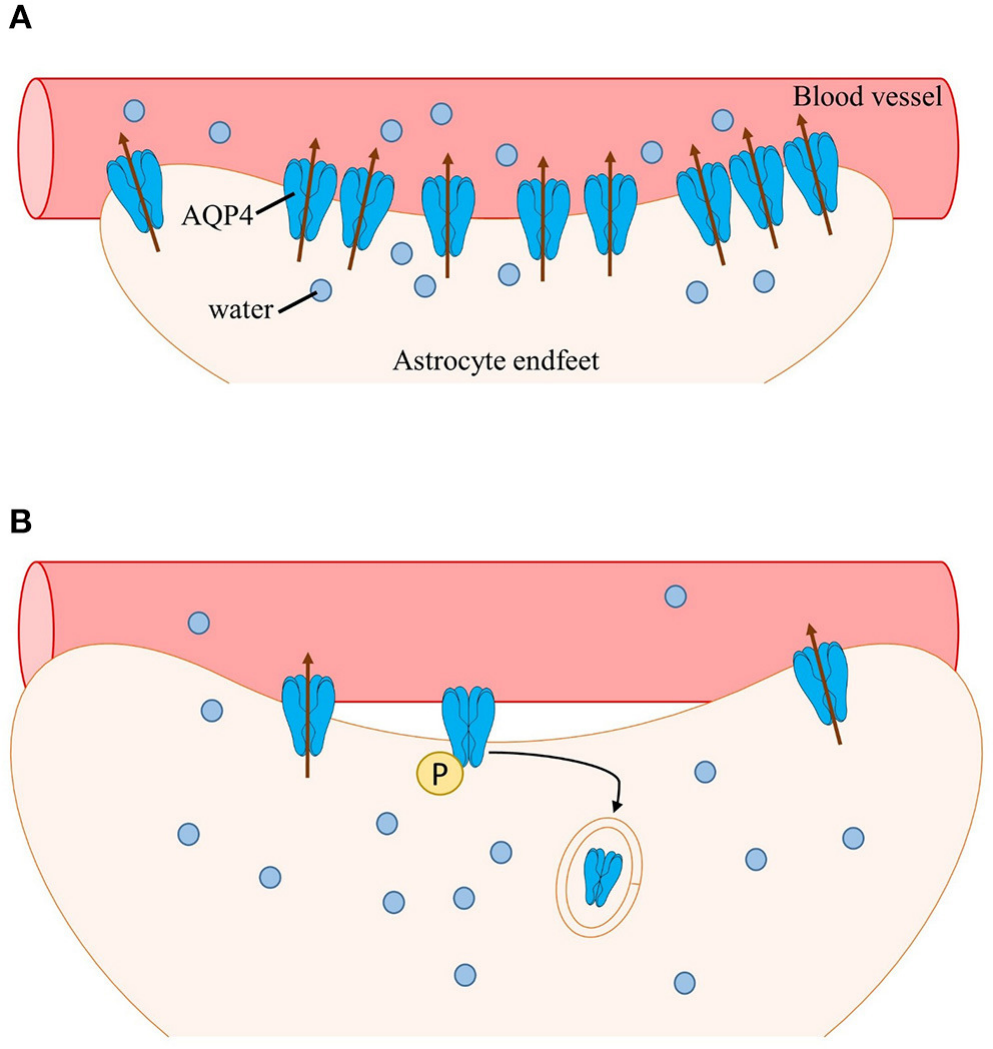
Schematic of AQP4 subcellular mislocalization. **(A)** After neuronal activation, water enters astrocytes (along with other ions and neurotransmitters such as K+ and glutamate). Water is then transported into nearby blood vessels via perivascular AQP4, overall maintaining water homeostasis. **(B)** During epilepsy, perivascular AQP4 (M23-AQP4) is lost due to loss of its anchoring protein α-syntrophin (not shown). Additionally, the rapid influx of water activates vasopressin receptors leading to phosphorylation of AQP4 by PKC followed by internalization and subsequent degradation of AQP4. Swelling of astrocytes occurs, resulting in impaired cell volume regulation and further increasing neuronal hyperexcitability.

Phosphorylation of AQP4 has been shown to play a role in subcellular localization (Nesverova and Törnroth-Horsefield, 2019; Vandebroek and Yasui, 2020). Early studies have shown that interaction of PKC activator phorbol 12-myristate 13-acetate (PMA) with AQP4 increases AQP4 phosphorylation leading to decreased osmotically-induced cell swelling (Han et al., 1998). Additionally, infusion of PMA in rat models of ischemia dramatically reduced brain water content by downregulating AQP4 (Okuno et al., 2008; Fazzina et al., 2010). Decreased AQP4 levels, which is correlated with an increased AQP4 internalization, is thought to be a result of AQP4 Ser180 phosphorylation by PKC. Moreover, AQP4 internalization induced by PKC activation was thought to be dependent on the binding of vasopressin to vasopressin 1_a_ receptors (V1_a_Rs) (Moeller et al., 2009). Rapid flux of water in the brain induced by neuronal activation has also been shown to be facilitated by activation of V1aRs (Niermann et al., 2001). Thus, the subcellular redistribution of AQP4 reported in clinical and preclinical findings may be via a PKC-dependent pathway. During intense neuronal activity, a large volume of water entering cells would activate vasopressin receptors; activation of vasopressin receptors, in turn, would trigger PKC phosphorylation of AQP4 and stimulate internalization and overall downregulation of AQP4 (presumably perivascular AQP4) (Figure 1).

## Conclusion

Taking into account the changes that AQP4 undergoes during epileptogenesis, it is important to examine how disruption of water homeostasis influences seizure activity. Subcellular mislocalization of AQP4 represents only one mechanism underlying the pathophysiology of epilepsy. However, it remains unclear whether this redistribution is a cause or consequence of the disease. It is clear that the loss of α-syn directly affects AQP4 polarization. The M1 and M23 isoforms of AQP4 were found to be have distinct functions and are differentially regulated during different phases of epilepsy. Posttranslational modifications of AQP4 driven by PKC activation may also alter AQP4 trafficking during epileptogenesis. Together, these findings suggest that AQP4 and/or its associated proteins can be possible therapeutic targets by either by upregulating perivascular AQP4 or preventing its relocalization.

It is important to note that transmembrane water fluxes in the brain is not solely regulated by AQP4, and thus swelling of astrocytes does not require AQP4 (Murphy et al., 2017b). Additional routes of water transport also rely on cotransporters such as Na^+^-K^+^-Cl^−^ (NKCC) (Macaulay et al., 2004; Kimelberg, 2005). In fact, high extracellular K+ stimulates NKCC1 leading to induction of astrocyte swelling and glutamate release (Su et al., 2002). Activation of NKCC1 is additionally enhanced by cell swelling thus promoting a positive feedback loop to increase further astrocyte swelling (Mongin et al., 1994). It is therefore not surprising that this cotransporter has been implicated in epilepsy where increased expression of NKCC1 was associated with the generation of seizures and epilepsy (Liu et al., 2020). Indeed, there are other molecules involved in astrocyte swelling (Kimelberg, 2005) which may also contribute to epileptogenesis.

Restoring water homeostasis may be a promising therapeutic strategy in certain contexts. For example, recent efficacy in treatment of CNS edema has been found by targeting the mechanism of calmodulin-mediated cell-surface localization of AQP4: remarkably, inhibition of calmodulin with the drug trifluoperazine (TFP) inhibited AQP4 mislocalization, ablated CNS edema and improved functional recovery after spinal cord injury (Kitchen et al., 2020). These findings were recently confirmed in a mouse model of photothrombotic stroke where treatment with TFP not only reduced CNS edema but also significantly decreased mRNA and protein levels of AQP4 during the acute phase of stroke. Most compelling was the effects of TFP on brain energy metabolism where an increased in glycogen was observed (Sylvain et al., 2021). Altered glycogen levels have been associated with seizures and epilepsy but with differing outcomes (Bak et al., 2018), therefore, treatment with TFP in epilepsy models would be most intriguing. It is conceivable that a similar approach to modulation of AQP4 may be therapeutic in preventing AQP4 mislocalization during epileptogenesis.

Although not discussed in this review, it is important to understand that complex molecular and signaling mechanisms of AQP4 exists and that their regulation is highly dynamic and intricate. For the purposes of novel drug discovery, one could explore the different steps involving AQP4 trafficking and recycling as well as the intracellular signals that modulate AQP4 activity (Markou et al., 2022; Salman et al., 2022b; Wagner et al., 2022). Moreover, gating mechanisms of AQP4 should also be considered, rather than mere redistribution. Indeed, it has been previously shown that AQP4 gating is dependent on specific conditions (Bernardi et al., 2018; Wei et al., 2022).

Due to its role in cell volume and ion regulation and its unique localization at fluid exchange barriers, AQP4 is involved not only in epilepsy but also in other neurological disorders such as cerebral edema, multiple sclerosis, Alzheimer's disease, and stroke. Therefore, research efforts to further elucidate the multifunctional roles of AQP4 during pathological states and the therapeutic potential of AQP4 regulation remain essential.

## Data Availability Statement

The original contributions presented in the study are included in the article/supplementary material, further inquiries can be directed to the corresponding author/s.

## Author Contributions

JS performed a complete literature review and drafted the manuscript. DB conceived of the manuscript and edited the manuscript. Both authors contributed to the article and approved the submitted version.

## Conflict of Interest

The authors declare that the research was conducted in the absence of any commercial or financial relationships that could be construed as a potential conflict of interest.

## Publisher's Note

All claims expressed in this article are solely those of the authors and do not necessarily represent those of their affiliated organizations, or those of the publisher, the editors and the reviewers. Any product that may be evaluated in this article, or claim that may be made by its manufacturer, is not guaranteed or endorsed by the publisher.
